# Close-Space Sublimation as a Scalable Method for Perovskite Solar Cells

**DOI:** 10.1021/acsenergylett.3c02794

**Published:** 2024-02-11

**Authors:** Nathan Rodkey, Inma Gomar-Fernández, Federico Ventosinos, Cristina Roldan-Carmona, L. Jan Anton Koster, Henk J. Bolink

**Affiliations:** † Instituto de Ciencia Molecular, Universitat de Valencia, Edificios Institutos de Paterna Calle Catedrático José Beltrán Martínez, 2, 46980 Paterna, Valencia, Spain; ‡ Zernike Institute for Advanced Materials, University of Groningen, Nijenborgh, 4, Groningen AE NL-9700, The Netherlands

## Abstract

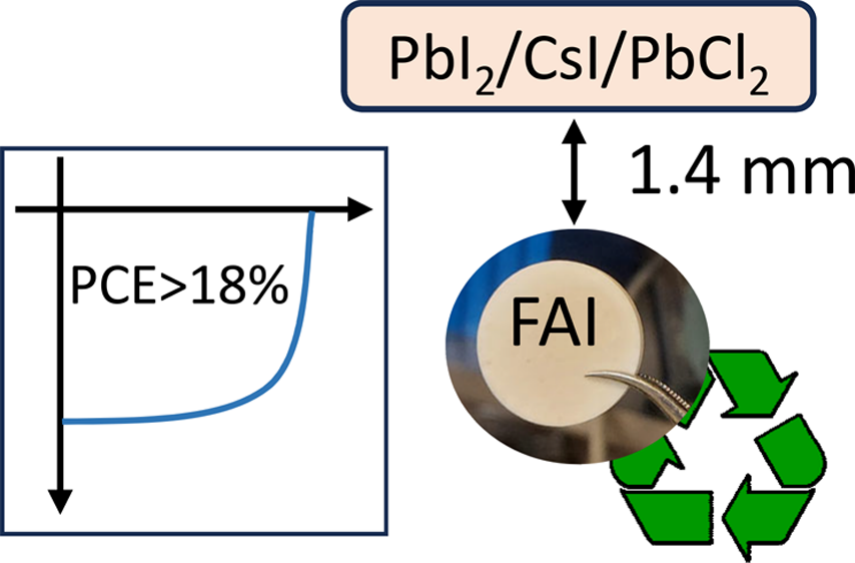

Vacuum techniques for perovskite photovoltaics (PV) are promising for their scalability but are rarely studied with techniques readily adaptable for industry. In this work, we study the use of close-space sublimation (CSS) for making perovskite solar cells, a technique that has seen widespread use in industry, including in PV, and benefits from high material-transfer and low working pressures. A pressed pellet of formamidinium iodide (FAI) can be used multiple times as an organic source, without needing replacement. Using CSS at a rough vacuum (10 mbar), efficient cesium formamidinium lead iodide perovskite based solar cells are obtained reaching a maximum photoconversion efficiency (PCE) of 18.7%. They maintain their performance for >650 h when thermally stressed at 85 °C in a nitrogen environment. To explain the initial rise in PCE upon heating, we used drift-diffusion simulations and identified a reduction in bulk trap density as the primary factor.

With increasing device performance in halide perovskites, the field has found itself in the challenging landscape of scalable manufacturing, especially in its application toward photovoltaics (PV), whose large-scale industrial manufacturing needs can be quite demanding. Furthermore, when targeting high-efficiency tandem configurations, silicon bottom-cells are often the choice candidate,^1−3^ requiring perovskites to keep up to the steep demands of the silicon PV industry when it comes to scale and speed. Current challenges in scalability and manufacturing of perovskites were summed up in several reviews, citing the instability of organic salts (such as methylammonium and formamidinium salts) and the choice of deposition technique as some of the key challenges.^1−8^

Halide perovskites are semiconductors that have the general crystal structure ABX_3_. Most efficient solar cells employ a mixture of A-site cations of which at least one is organic in nature, such as formamidine (FA). Small-area perovskite solar cells are typically processed by using solvent-based methods such as spin-coating. However, solvent-free methods also exist to prepare halide perovskites, with the co-sublimation of perovskite precursors in a high-vacuum chamber one of the most reported of these techniques.^9,10^ High-efficiency and stable PV devices have been prepared using this co-sublimation method on both small- and large-area devices.^6−8,11^ Even though co-evaporation in high-vacuum can be scaled to large areas, as is done for commercial OLEDs,^12^ there are some drawbacks to this method. These include the need for high-vacuum (<1 × 10^–5^ mbar), in situ sublimation monitoring to control the stoichiometry of the deposited film, and rotation of the substrates to ensure homogeneity over large areas. Furthermore, the organic sources used (e.g., FAI and MAI) tend to be unstable, decomposing over time. An alternative high-vacuum-based process relies on the formation of the perovskite in two consecutive steps. In this two-step (e.g., sequential) deposition method the inorganic perovskite precursors are first sublimed onto a suitable substrate using a moderate to high vacuum. The organic A-site cation is then deposited in a second step on the inorganic film (sometimes referred to as a scaffold), which leads to the formation of the perovskite. In most works, the A-site cation is deposited using a solvent-based step,^13−19^ but a few reports also exist that deposit the A-site cation using a second sublimation step.^20−22^ In most sequential deposition processes, an annealing step is required to (fully) convert the precursors into the perovskite structure. Such a two-step method employing thermal evaporation of precursors was shown recently in a record dry-vacuum process by Li et al., who reported a 24% efficient device.^23^ Close-space sublimation (CSS) was explored as early as 2016^16,22^ for the preparation of perovskites in vacuum. CSS is a variety of sequential deposition techniques in which both steps are carried out in a vacuum but in which at least one of the sublimation processes is carried out using a close-space sublimation tool. In such a tool, the distance between the substrate and the source containing the sublimable materials is kept small (a few millimeters at most). The substrate is often kept at an elevated temperature, and the perovskite is formed during the second step without the need for an additional annealing step. This is atypical from most reports for two-step sublimation processes (often employing high-vacuum thermal evaporation chambers and/or spin-coating) who show the formation of a double layer, or partially formed layer, which is converted to the perovskite by a subsequent annealing step.^23,24^

Compared to co-sublimation, CSS processes have some notable advantages. For one, by reducing the distance between the substrate and source, the vacuum requirements to maintain a sufficiently long mean-free path of sublimated materials is reduced. This allows the use of rough vacuum (>1 × 10^9^mbar), with CSS working pressures typically reported between 1 and 100 mbar. This reduced substrate-to-source distance is accompanied by a high degree of material-transfer, with little waste or lost materials during the process. Additionally, by using a sequential processing method, the need for in situ rate monitoring is removed, and the complexity of the system is reduced. While most CSS reports focus on the sublimation of the organic precursors, some groups have also shown that sublimation of inorganic precursor layers by CSS is possible, pointing to the potential of in-line CSS processes that sublime both the inorganic and organic components of the perovskite solar device.^20,21^

CSS processes have reached efficiencies as high as 21.27%, reported by Tie et al.,^18^ and similar works converting films without an additional annealing step have reached efficiencies as high as 22.06% reported by Hu et al.^25^ However, their use of spray-coated glass for the organic source hampers the incorporation of such a system into an industrial process line, instead pushing it toward batch-processing and limiting its potential for scalability. Despite this, nearly all high-efficiency works related to CSS (>17%) rely on solvent-based processes (i.e., spray- or spin-coating) to deposit the organic A-site cation onto a substrate so that it can be heated in the CSS tool as the organic source.^14,17,18,25,26^ These sources are single-use as the organic is deposited by spin- or spray-coating, after which new organics must be redeposited between each conversion step. However, by use of powders or pressed pellets, organic sources can potentially be reused many times. In this work we demonstrate the solvent-free preparation of FA_0.9_Cs_0.1_PbI_3_:Cl perovskites by converting an inorganic precursor layer in a custom-built CSS setup. This is done with a reusable organic source (FAI), independent control of substrate and source temperatures (120/150 °C), and a low working pressure (10 mbar). Using this method and tool we were able to obtain efficient, fully vacuum-processed perovskite solar cells. The champion device had a photoconversion efficiency (PCE) of 18.7%. We describe in detail the process optimization needed to obtain these high efficiencies. Furthermore, we analyze the cell performance over time at elevated temperatures with the aid of drift-diffusion simulations. This analysis points to a reduction in trap density (*n*_T_) by 5 orders of magnitude after 8 days at 85 °C.

## Organic Source Reusability and Thin-Film Properties

**Figure 1:**
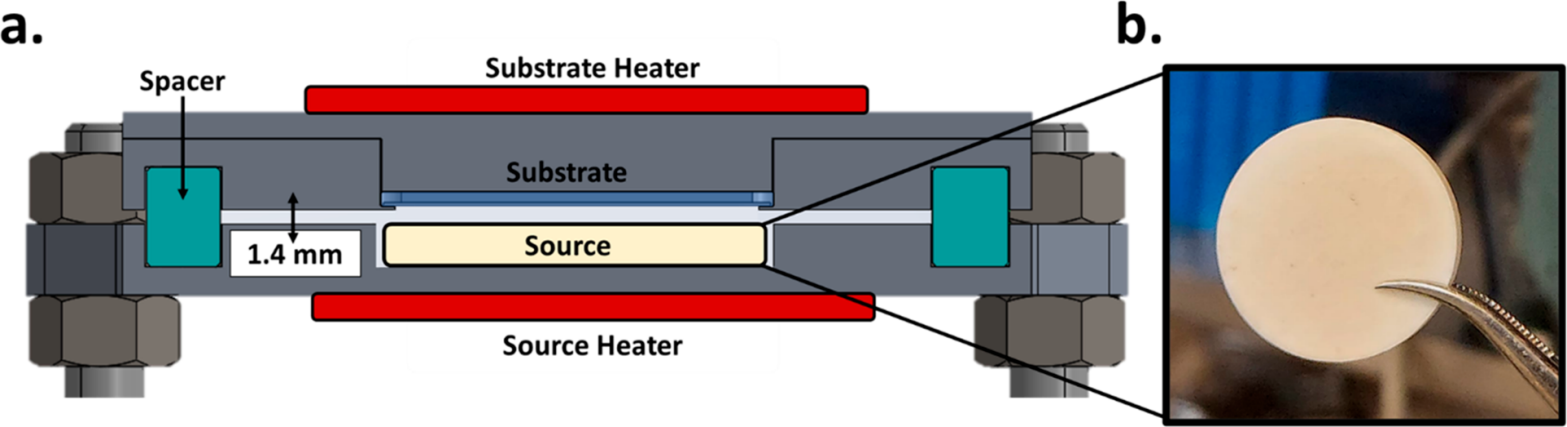
(a) Solidworks cross section of the close-space sublimation chamber used in this work. The major components are labeled: substrate heater, source heater, substrate, and source. Notably, a 1.4 mm gap is maintained between the source and the substrate using ceramic spacers. (b) An example of the pressed organic pellets used in this work (in this case FAI).

The CSS system used in this work is shown in further detail in Figure 1a, where a cross-sectional image produced from Solidworks can be seen. In Figure 1b, a pressed pellet of FAI powder is shown used as the organic source. The pellet itself is a 20 mm diameter, 3 mm thick cylinder made using a hydraulic press by applying a pressure of 300 MPa for 30 min. XRD patterns of FAI powder before and after pressing are shown in Figure S1, showing little change in the diffraction. This pellet went into the lower part (source) of the CSS chamber, where a 3 mm indentation ensures that the pellet is level with the surface of the source plate (Figure S2). Thus, a working distance of 1.4 mm can be well-defined using ceramic spacers (and considering the shelf and shadow mask upon which the sample sits). Once the chamber is sealed and a working pressure of 10 mbar of N_2_ is set, we start to heat the substrate, and afterward the source, by ramping them at 15 °C/min. Note, both the source and substrate have independent heating control. In CSS, the parameters that control the deposition are the substrate and source temperatures and the duration of the process. This duration, or conversion time, is defined in this work as the time from when the source reaches the target temperature until the moment the heaters are turned off. The substrate/source temperatures used in this work were 120/150 °C with conversion times varied from 10 to 30 min. These temperatures were chosen after an initial screening of substrate/source temperatures where we identified a promising temperature window, which was subsequently used to investigate in detail the conversion of an inorganic scaffold (Figure S3).

**Figure 2:**
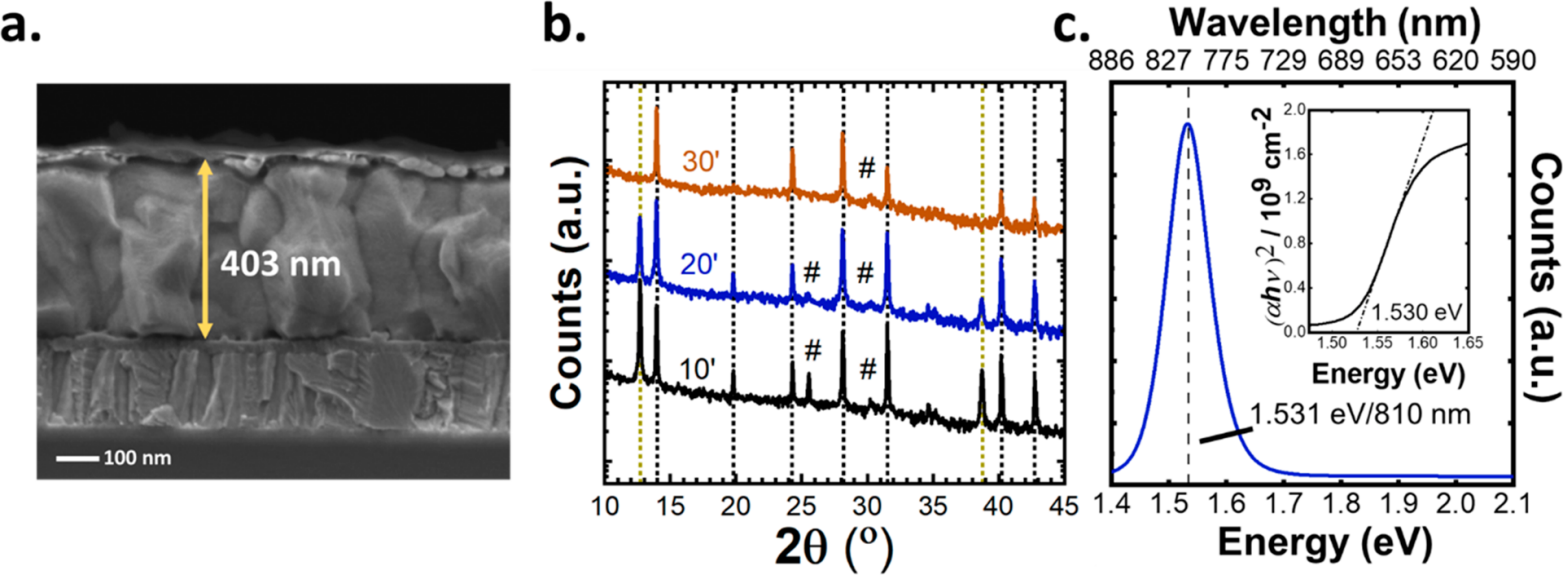
(a) SEM cross section of a completed device, where it can be observed that grains are on the order of the film thickness (∼400 nm). (b) XRD of inorganic precursor layers converted for 10, 20, and 30 min. The reduction of a PbI_2_ peak is apparent at 12.7°. The optical properties of these films were verified in panel c through PL and Tauc analysis for direct bandgap materials. This analysis showed a PL peak emission at 1.531 eV and a Tauc bandgap at 1.530 eV.

**Figure 3:**
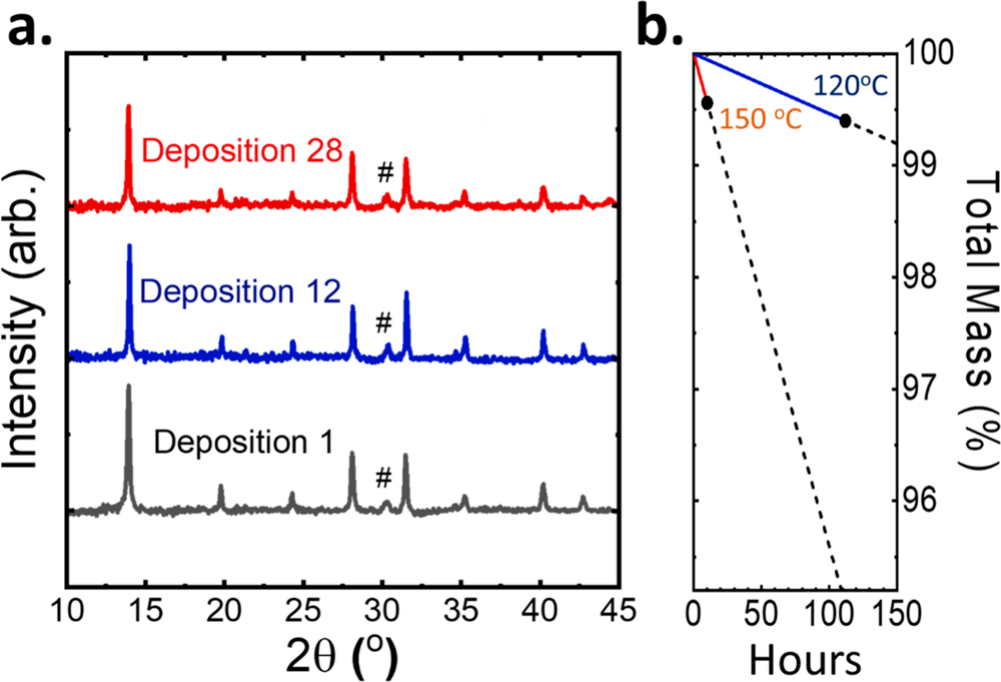
Reusability of an FAI source was verified through the repeated conversion of inorganic precursor layers by employing the same FAI source. Films were converted for 30 min, with the substrate/source kept at 120/150 °C. (a) XRD patterns of depositions 1, 12, and 28 converted in this way. Marked by a pound (#) sign is a diffraction peak originating from the ITO substrate. The mass of pellets was measured after repeated cycling and thermal stressing, shown in panel b for a source temperature of 150 °C (red) and 120 °C (blue). This thermal stressing was done in a static vacuum of 10 mbar and a substrate temperature of 120 °C. Detailed information is shown in Table S1, where an FAI pellet kept at 120 °C for 112 h lost 0.66% of its total mass. In dashed lines, a linear extrapolation of %mass/hour is shown.

**Figure 4:**
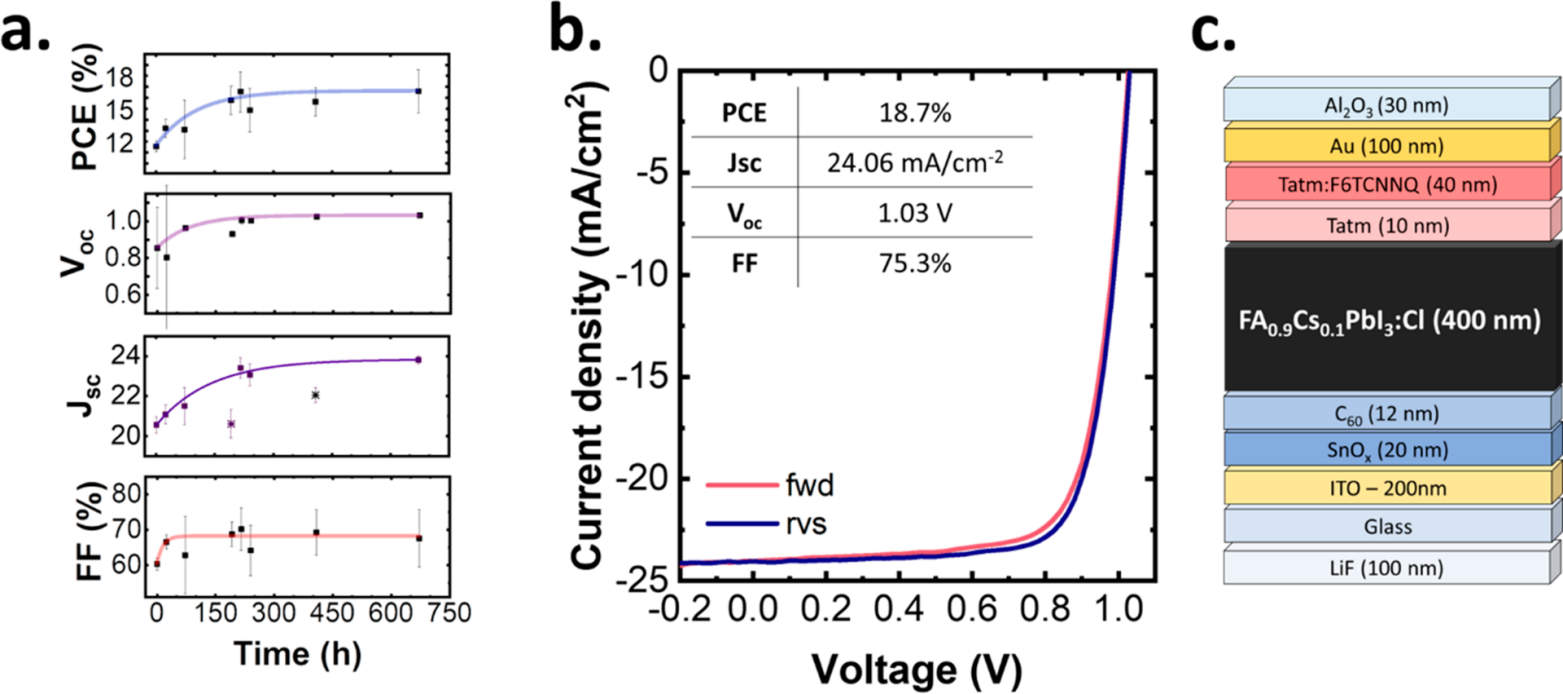
CSS devices were studied over prolonged annealing at 85 °C. (a) Stability of the PCE, *V*_oc_ (V), *J*_sc_ (mA/cm^2^), and FF (%). They are proven to be quite stable after >650 h annealing. Trendlines were fit by a monoexponential decay and include all points with the exception of the *J*_sc_, where two outliers are observed (marked as stars). (b) Forward and reverse *J*–*V* curves of a champion device are shown reaching a PCE of 18.7%. This champion efficiency was achieved after 8 days on the hot plate in open-circuit conditions and under ambient lighting conditions. (c) Device architecture. Notably, we employ a nip archtiecture with SnO_*x*_/C_60_ as the bottom contact due to the elevated temperatures involved in the conversion process.

We selected a nip stack for our initial studies with the CSS tool. The electron extraction layers that are used on top of an ITO coated glass substrate are SnO_*x*_ and C_60_ (in that order), are thermally stable, and will allow heating of the substrate to temperatures as high as 200 °C. The SnO_*x*_ films were deposited using ALD and the C_60_ using thermal sublimation in a high-vacuum chamber. The layer thicknesses of both films were 20 and 12 nm, respectively. A 210 nm layer of inorganic perovskite precursors (e.g., inorganic scaffold), PbI_2_, PbCl_2_, and CsI were also deposited using a high-vacuum thermal sublimation, with the relative ratio controlled via coevaporation, as described in detail in Experimental Methods. These films were then loaded into the CSS system. Subsequently, the conversion process of a FA_0.9_Cs_0.1_PbI_3_:Cl (10%) perovskite was investigated by a combination of SEM, XRD, and PL (shown in Figure 2). In Figure 2a it can be seen from the SEM cross-sectional image of a completed device (converted for 20 min) that the grains are large (∼400 nm) and on the order of the film thickness. This 20 min conversion was chosen as it was observed to be the optimal conversion time for working devices (Figure S4 and as reported later in Figure 4). While devices reported in this work were optimized for 400 nm, the potential for the sequential conversion of thick films by this technique is shown in Figure S5 where >1 μm thick films with large grains are seen, with no residual PbI_2_ peaks in its corresponding XRD pattern. In Figure 2b, the XRD pattern for conversion times of 10, 20, and 30 min are shown. Notably, the disappearance of PbI_2_ diffraction peaks at 12.7° and 38.9° (yellow dotted lines) is seen for longer conversion times. Marked by a pound sign (#) are the diffraction peaks from the ITO substrate, and the black dotted lines indicate the diffractions attributed to a cubic perovskite structure. In Figure 2c the photoluminescence (PL) spectrum of the FA_0.9_Cs_0.1_PbI_3_:Cl (10%) perovskite converted for 20 min in the CSS setup is shown. The peak PL positioned at 810 nm (1.53 eV) compares well with that calculated from a direct bandgap Tauc analysis based on the absorptance spectrum of a 1.53 eV (inset). This represents a shift to higher energies from that of pure α-phase FAPI reported at 1.48 eV which correlates to the addition of Cs and Cl (both 10% molar) added to the inorganic precursor layers which both expand the lattice and push the bandgap toward higher energies.^27^ To ensure the reusability of the organic FAI source, we tracked the X-ray diffraction (XRD) of converted perovskite films over repeated deposition cycles using the same experimental conditions (10 mbar working pressure, 120/150 °C substrate/source temperatures, 30 min conversion times). The XRD pattern does not change significantly over 28 deposition cycles, shown in Figure 3a with black dashed lines marking the perovskite diffraction peaks and a pound sign (#) indicating those of the substrate. The weight loss of the FAI pellet used in these repeated cycles was measured after 28 depositions. Starting from a weight of 2263 mg, only 11 mg (0.49%) of the FAI had been lost during these depositions, or 0.0175% per deposition. The 28 deposition cycles imply a total of 10 h of sublimation at 120/150 °C (substrate/source) at a working pressure of 10 mbar, capable of converting a combined layer thickness of 9 μm of inorganic perovskite precursor films. A separate FAI pellet was stressed for 112 h at a substrate/source temperature of 120/120 °C, in a static vacuum of 10 mbar. This information is listed in Table S1 and seen visually in Figure 3b. Marked in dashed lines is the linear extrapolation of the continued mass loss. After 112 h, the FAI pellet lost 0.61% of its total mass. This low mass loss and ability to repeatedly cycle the FAI target is contrary to problems reported by other groups related to degradation of the FAI precursor in thermal sublimation or gasflow sublimation works^28−30^ and points to the promise of CSS in an industrial process line, where sources are often used for months at a time. Notably, sublimation and conversion of the inorganic precursor layers is observed at these temperatures (Figure S6). At the substrate/source temperatures of 120/120 °C the inorganic layer is not completely converted as evidenced by the residual diffraction peak of PbI_2_. Our setup allows one to individually control the substrate/source temperatures, and as mentioned before when using a substrate/source temperature of 150/130 °C, full conversion was achieved even for films as thick as 1 μm (Figure S5).

Using the optimized FA_0.9_Cs_0.1_PbI_3_:Cl (10%) process, photovoltaic devices were fabricated using SnO_*x*_ (20 nm)/C_60_ (12 nm) as the ETL and Tatm (10 nm)/ Tatm:F6TCNNQ (40 nm) as the HTL. Later, a 100 nm LiF antireflection coating was thermally evaporated onto the glass side. The device was finished with 100 nm of Au contacts and encapsulated by Al_2_O_*x*_ using atomic layer deposition (ALD). Detailed information on device fabrication, characterization, and materials is included in Experimental Methods.

## Device Stability

The initial PCE of the champion device was 12.2%; however, upon thermal stressing, it improved to 18.7%, attributed to a reduction in bulk and surface defects detailed more thoroughly in the subsequent section. The devices were stressed at 85 °C in a nitrogen atmosphere, which increased the average device PCE from 11.6 to 16.6% over 8 days. Interestingly, a short annealing process of 100 °C for 2 min noticeably improved device performance (Figure S7) and may point to the feasibility of shorter annealing times for improving device performance. Despite this, a prolonged annealing at 85 °C was chosen for this study to compare with an existing baseline for thermal stressing of perovskite solar cells in our group.^31^ Device stability and performance were tracked for a total of 650 h. In Figure 4a, the notable increase in PCE occurs over the first 200 h and is reflected in the FF, *J*_sc_, and *V*_oc_ where trend lines have been marked. In particular, the *V*_oc_ improved from 0.8 to 1.04 V over 9 days of thermal stressing. Further information on the normalized PCE of the individual devices is shown in Figure S8. The *J*–*V* forward and reverse scans of a champion device are shown in Figure 4b with the device architecture used in Figure 4c. This champion efficiency was observed on day 8 (216 h). Hysteresis across the devices were minimal with the average PCE for the 8 devices reported on this day at 16 and 16.55% (Figure S9) for forward and reverse scans, respectively.

## Drift-Diffusion Simulations

**Figure 5:**
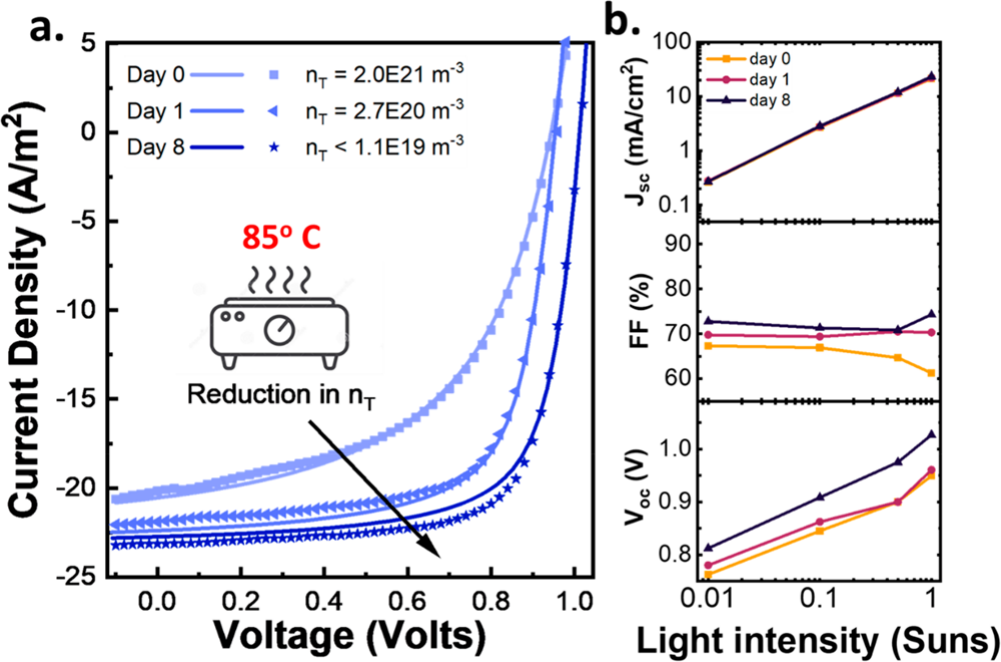
*J*–*V* characteristics of a CSS sample were tracked throughout the annealing/stressing process. (a) Measured reverse *J*–*V* sweep (scatter) and simulated sweep (solid lines). Simulated *J*–*V* curves were done using a drift-diffusion simulation software (SIMsalabim)^32^ described in greater detail in the main text. The observed increase in performance is prescribed to a reduction in bulk trap density (*n*_T_), going from 2.0 × 10^21^ to 1.1 × 10^19^ traps/m^–3^. This sample was tracked on day 0 (as deposited), day 1, and day 8 of thermal stressing on a hot plate at 85 °C in a nitrogen atmospher. (b) Intensity-dependent *J*–*V* characteristics. The constant fill factor over light-intensity is an indication that shunt and series related losses are minimal.

To better understand the nature of the improvement seen after prolonged annealing/stressing, drift-diffusion simulations were carried out using an open-source code, SIMsalabim.^32^ In Figure 5, the reverse *J*–*V* scans of a sample as deposited (day 0), annealed for 1 day, and annealed for 8 days are plotted side-by-side (solid lines). The simulated *J*–*V* characteristics are then plotted with symbols. In the simulation, the bandgap was fixed using a Tauc analysis for direct bandgaps shown in Figure 2, and parameters for the transport layers were taken from a previous publication of this group.^33^ Similarly, starting values for electron/hole mobilities, series/shunt, interface defect, and bulk trap (*n*_T_) densities were taken from this previous simulation work. This initial seed allowed for a fit of day 8 with <2% RMS, using an autofit feature provided in the open-source code, and while allowing for fluctuations in all the aforementioned parameters. RMS values were calculated using eq S1. With this good initial fit of day 8, we used it as a seed for both days 0 and 1, investigating which parameters had the most dramatic impacts on the quality of fit. Following a hypothesis that the low *J*_sc_ observed on day 0 and day 1 were related to defect states, we allowed 5 related parameters to vary during the fitting process: μ_n0_, μ_p0_, *S*_tn_, *S*_tp_, and *n*_T_. Where μ_n0_ and μ_p0_ are the electron and hole zero-field mobilities, *S*_tETL_ and *S*_tHTL_ are the interface trap densities at the ETL and HTL, and *n*_T_ is the bulk trap density. Additionally, to obtain trap-relevant device parameters, we chose to fit the series (*R*_series_) and shunt (*R*_shunt_) resistance. We found that *n*_T_ had the largest impact on fit quality, where large differences (several magnitudes) were needed to achieve low RMS values, seen in Table S2 where the fitted device parameters for days 0, 1, and 8 are shown. The full descriptions of these simulated *J*–*V* curves are provided in the Supporting Information as txt files for all 3 days. In Figure S10 we corroborate that this change comes from the bulk trap density, showing that when this parameter is incremented, while allowing for interface trap densities and carrier mobilities to fit over a wide range, RMS values never approach those of the initial fit. In Figure S10 a minimum RMS value is seen for day 0 and day 1, while for day 8, a plateau is observed at lower bulk trap densities suggesting they no longer limit the device performance, instead likely limited by interface traps. As such, for day 8, while we simulate a decrease in trap density, we specify the bulk trap density as a range <1.1 × 10^19^ m^–3^ since equivalent RMS values can be achieved for even lower bulk trap densities. In the end, the simulated bulk trap densities were 2.0 × 10^21^, 2.7 × 10^20^, and <1.1 × 10^19^ m^–3^ for days 0, 1, and 8 respectively. This prescribes the drastic improvement in device performance to a reduction in bulk trap density of ∼2 orders of magnitude. Normalized RMS values for each *J*–*V* curve are <2%. A noticeable hysteresis is observed in Figure S11 in the *J*–*V* curves of as-deposited devices (day 0), which disappeared after prolonged annealing, an indication of trap-assisted ion migration.^34^ Additionally, the light intensity *J*–*V* parameters are shown in Figure 4b. The fill factor, which is relatively independent of the light-intensity, suggests that shunt and series losses do not play a major role, instead dominated by bulk and/or interface recombination.^35^

Using a custom-built close-space sublimation chamber (CSS) we have prepared fully evaporated nip Cs-FA-Pb-I-based perovskite PV cells, reaching a maximum efficiency of 18.6%. An inorganic scaffold consisting of Cs–Pb–I–Cl prepared by high vacuum co-sublimation was converted to the FA-containing perovskite in a time frame of 20 min. A pressed pellet of FAI was used as a dry and reusable source. Mass loss in the source pellet was tracked and found to be a small fraction (0.0175%) per deposition, and it was used for 28 subsequent depositions. This highlights the compatibility of the pressed FAI source with continuous operation at the sublimation temperatures. Thus, obtained PV devices improve significantly on prolonged heating at elevated temperatures (85 °C). Using drift-diffusion open-source software (SIMsalabim) we were able to fit the experimental *J*–*V* curves by varying primarily the trap density as the fitting parameter. This indicates that a reduction of bulk traps upon annealing leads to an increase in device performance. We believe the results of this work offer a promising outlook for CSS for halide perovskite solar cells.

## Experimental Methods

Prepatterned ITO coated glass substrates were purchased from Naranjo substrates. These substrates were cleaned in subsequent sonicated baths of soap, deionized water, and isopropyl alcohol. After which they were placed in an atomic layer deposition (ALD) system (Arradiance’s GEMStar XTThermal) for the deposition of SnO_*x*_. The SnO_*x*_ layer thickness was 20 nm and was amorphous after deposition. The substrates were then annealed at 150 °C for 30 min. These substrates were then transferred under inert conditions to a high-vacuum sublimation system (∼1 × 10^–6^ mbar) for the deposition of the C_60_ layer (12 nm). The deposition rate was controlled using a quartz crystal microbalance (QCM). The now ITO/C_60_/SnO_*x*_ coated glass was transferred to a second high-vacuum sublimation chamber (∼1 × 10^–6^ mbar) for sublimation of the inorganic perovskite precursors. This inorganic layer consists of 200 nm of coevaporated PbI_2_, CsI, and PbCl_2_. The molar ratio of Cs and Cl in the perovskite structure was kept to 10% using the equation *N* × *c*/*M* where *N* is the film thickness, *c* is the density, and *M* is the molecular weight. The control of ratios and thickness was done via QCM.

Next, the sequential deposition of FAI by close-space sublimation was done at a working pressure of 10 mbar, substrate/source heating of 120/150 °C, and a separation between the source and substrate of 1.4 mm. This spacing comes from a gap defined by ceramic spacers (1 mm) as well as the shelf (0.2 mm) and shadow mask (0.2 mm) upon which the substrate rests. A Solidworks cross section of the close-space sublimation chamber used in this work is provided in Figure 1a. Notably, the source and substrate heaters can be controlled independently. Traditional close-space sublimation chambers rely on the use of moving substrates as an effective shutter for their sublimation processes, as this was impractical to the design of a small-area tool, where the precursor film is continuously exposed to the source material. For process reliability we chose to first heat the substrate to the target temperature, before ramping the source temperature at 15 °C/min. The conversion times reported here start once the target source temperature is reached. An FAI pellet was used as the source by pressing powder into a 20 mm diameter, 3 mm thick cylindrical pellet (Figure 1b). This was done at a pressure of 300 MPa for 30 min. To track the reusability of the FAI pellet, it was first massed before being loaded into the sublimation chamber.

After conversion of the inorganic scaffold by FAI deposition in the CSS, TaTm (10 nm) and TaTm with F6TCNNQ (40 nm) were deposited as the hole transport layers. The doped layer was done by coevaporating TaTm and F6TCNNQ with rates of 0.8 and 0.1 Å/s, respectively. The full molecular description of TaTm is ((N4,N4,N4″,N4″-tetra([1,1′-biphenyl]-4-yl)-[1,1′:4′,1″-terphenyl]-4,4″-diamine), and F6TCNNQ is 1,3,4,5,7,8-hexafluorotetracyanonaphthoquinodimethane. Finally, the devices were finished with evaporated Au contacts and Al_2_O_*x*_ encapsulation via ALD.

For device characterization, a Wavelabs Sinus 70 AAA LED solar simulator was used (in air). The illumination intensity for 1-sun was calibrated to AMG1.5 using a calibrated Si reference diode equipped with an infrared cutoff filter (KG-5 Schott). A mismatch factor of 1.01 was calculated using the spectral irradiance of AMG1.5 and the LED simulator compared to the EQE of the reference cell and a FA_0.9_Cs_0.1_PbI_3_:Cl (10%) device, provided in Figure S12. Devices had a total area of 0.0825 cm^2^ and an illuminated area of 0.05 cm^2^, defined using a shadow mask during both the deposition and measurement process. Photoluminescence was measured in the steady state using a 522 nm green excitation laser. X-ray diffraction was measured with a powder diffractometer Empyrean from Panalytical, equipped with a Cu Kα anode operated at 45 kV and 40 mA. For capillary measurements, powder was prepared in a 1 mm tube, sealed in a nitrogen glovebox, and kept in constant rotation during the measurement. SEM images were taken with a field emission scanning electron microscope (HR-FESEM), ZEISS GeminiSEM 500 model, with a secondary Electron In-Lens detector using an accelerating voltage of 0.7–1 kV.

## Supporting Information Available

Additional XRD files, cross-sectional SEM images, photographs of the FAI pellet and CSS system, statistical information regarding the solar cells performance (PDF)
Additional .txt files: “day 0.txt”, “day 1.txt”, and “day 8.txt” contain parameters used for drift-diffusion simulations corresponding to days of annealing at 85 °C as referred to in the main text (ZIP)
