# Supplementary material for: Close-Space Sublimation as a Scalable Method for Perovskite Solar Cells
Source: ACS Energy Lett. 2024 Feb 11;9(3):927–33. doi: 10.1021/acsenergylett.3c02794 (PMC10928705; doi:10.1021/acsenergylett.3c02794)
Supplement: Supplementary file 1 — nz3c02794_si_001.pdf [file nz3c02794_si_001.pdf]

Close-Space Sublimation as a Scalable Method for Perovskite Solar Cells

Nathan Rodkey[1], Inma Gomar-Fernández[1], Federico Ventosinos[1], Cristina Roldan-Carmona[1], L. Jan Anton Koster[2], Henk J. Bolink[1,*]

[1]*Instituto de Ciencia Molecular – Universitat de Valencia*
Edificios Institutos de Paterna Calle Catedrático José Beltrán Martínez, 2, 46980 Paterna, Valencia

[2]Zernike Institute for Advanced Materials, University of Groningen
The Netherlands, NL-9700, AE Groningen, Nijenborgh, 4

**henk.bolink@uv.es*

Normalized RMS values are reported in this work as a way of quantifying the deviation between the simulated and measured JV curves, calculated using **Eq. S1**:

$$r = \frac{\sqrt{\sum_i (J_i^{(sim.)} - J_i^{(meas.)})^2}}{[\max\ (J^{(sim.)}, J^{(meas.)}) - \min(J^{(sim.)}, J^{(meas.)})]'} \qquad \textbf{Eq. (S1)}$$

Where J_i denotes the current density, sim. and meas. the simulated and measured JV curves respectively, and r the normalized RMS.

$$\mathrm{M} = \frac{\int_{\lambda_1}^{\lambda_2} E_{AMG1.5}(\lambda) S_{ref}(\lambda) d\lambda}{\int_{\lambda_1}^{\lambda_2} E_{AMG1.5}(\lambda) S_{sample}(\lambda) d\lambda} \frac{\int_{\lambda_1}^{\lambda_2} E_{Lamp}(\lambda) S_{sample}(\lambda) d\lambda}{\int_{\lambda_1}^{\lambda_2} E_{Lamp}(\lambda) S_{ref}(\lambda) d\lambda} \qquad \textbf{Eq. (S2)}$$

Above, an equation for calculating mismatch factor (M) using the EQE of a reference simple and calibrated diode as well as the irradiance of a solar simulator compared to AMG1.5. Above, E represents the irradiance and S the spectral response. The mismatch calculated in this work was 1.01.

Table S1. Pressed pellets of FAI were used as organic sources for the conversion of inorganic precursor layers. They were used multiple times, and their mass was tracked below to gauge their longevity. At both substrate/source temperatures of 120/150 ºC and 120/120 ºC conversion of inorganic precursor layers has been observed **Fig. SX**. For the 120/120 ºC thermal stress for 112 hours, a mass loss of 0.61% was recorded.

Starting Mass (mg)	Substrate/ Source (ºC)	Working Pressure (mbar)	Mass loss	Time (h)	Inorganic Precursor Total (µm)	# Samples
2263	120/150	10	11 mg/0.49%	10	9	28
2147	120/120	10	13 mg/0.61%	112	-	-

Table S2. Table of fitted device parameters used during drift-diffusion simulations for days 0, 1, and 8. The largest difference is seen in the bulk trap densities which decrease by ~2 orders of magnitude after 8 days of thermal stressing/annealing in a nitrogen environment.

Parameter	Symbol	Day 0	Day 1	Day 8
zero-field mobility of electrons	μ_{n0}	3.071E-5	4.592E-5	1.795E-5
zero-field mobility of holes	μ_{p0}	2.005E-5	1.935E-5	2.773E-5
bulk trap density	n_T	1.97E21	2.702E20	1.075E19
interface trap density at ETL	S_{tETL}	1.410E16	5.333E15	1.004E16
interface trap density at HTL	S_{tHTL}	2.037E15	9.629E14	1.876E14
series resistance	R_{series}	3.497E-6	1.35E-4	3.884E-5
shunt resistance	R_{shunt}	1.254E1	1E2	4.512E1

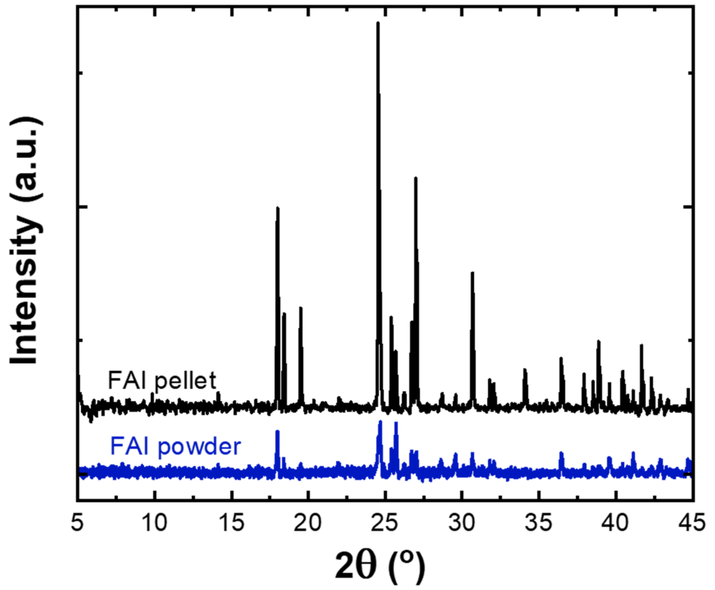

Figure S1. Powder x-ray diffraction of FAI powder (blue) and a pressed pellet (black) are shown above. FAI powder was measured in a capillary tube, with powder loaded in a nitrogen, humidity-free environment as described in the *Experimental Methods*. The FAI pellet was both pressed and measured in air, where exposure to water and oxygen may have affected the measurement (as seen by the additional peak near 34°). Besides the unexpalined peak at 34°, the spectrums match well, indicating little change between the powder and pressed forms.

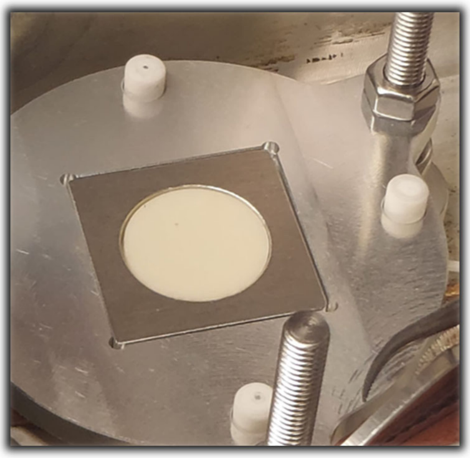

Figure S2. A photo of a pressed FAI pellet, loaded into the close-space sublimation system is shown. A thickness of 3 mm ensures that the surface of the pellet is level with the metallic backplate. Seen as well is a square mask that centers the pellet to the substrate above. In this way, the working distance between the source and substrate can be defined by the ceramic spacers (seen on the edges as white pillars), while also considering the thickness of the shadow mask and shelf upon which the substrates lay. The distance defined here is a 1.4 mm coming from the ceramic spacers (1 mm), the shelf upon which the sample lies (0.2 mm), and the shadow mask (0.2 mm).

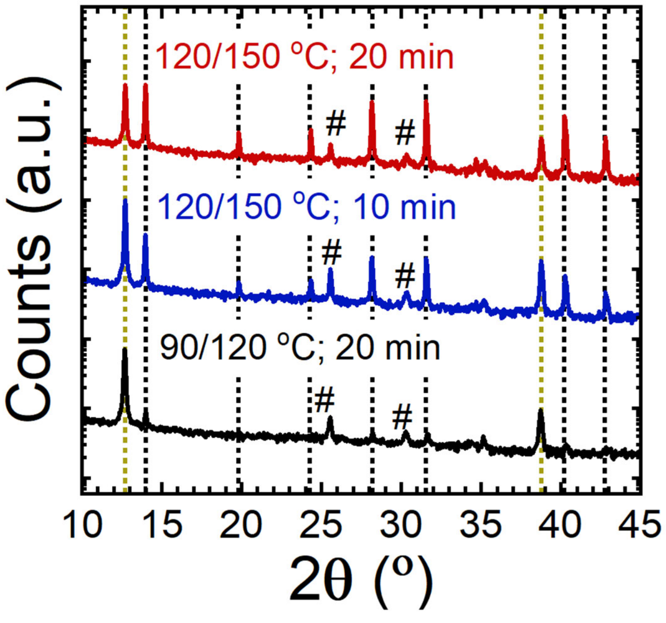

Figure S3. X-ray diffraction of samples converted with different substrate/source temperatures and conversion times are shown above. Inorganic scaffolds of PbI_2, $PbCl_2$, and CsI were converted using a pressed FAI pellet as a source, as described in the *Experimental Methods*. In black, a sample using a substrate/source temperature of 90/120 °C shows a strong PbI_2 peak (yellow-dashed lines, note the logarithmic scale), indicating that at 120 °C the FAI does not show significant sublimation. Moving to temperatures of 150 °C shown in blue and red, perovskite diffraction peaks become more prominent (shown in black-dashed lines).

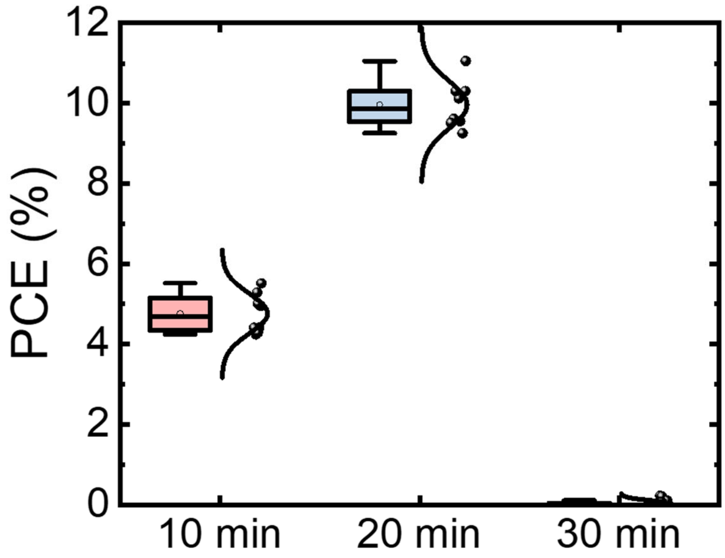

Figure S4. Device performance of samples converted for 10, 20, and 30 minutes were evaluated. The sensitivity to the process to conversion time is evident, with samples converted for 20 minutes displaying the highest initial efficiencies.

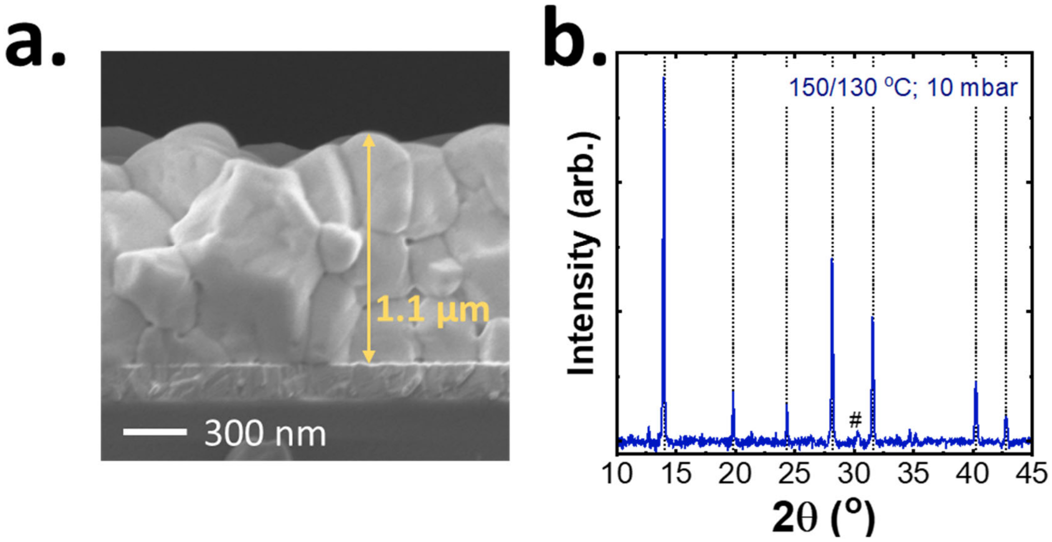

Figure S5. Thick (650 nm) inorganic precursor layers composed of $CsI/PbI_2/PbCl_2$ were loaded into the CSS system and converted as described in the *Experimental Methods* section. A working pressure of 10 mbar was used, converting for 50 minutes, at a substrate/source temperature of 150/130 ºC. In **a.** the cross-sectional SEM showing large grains and >1 µm thickness and in **b.** the corresponding XRD pattern, where the pound (#) sign marks diffraction the ITO substrate and dotted black lines the perovskite peaks.

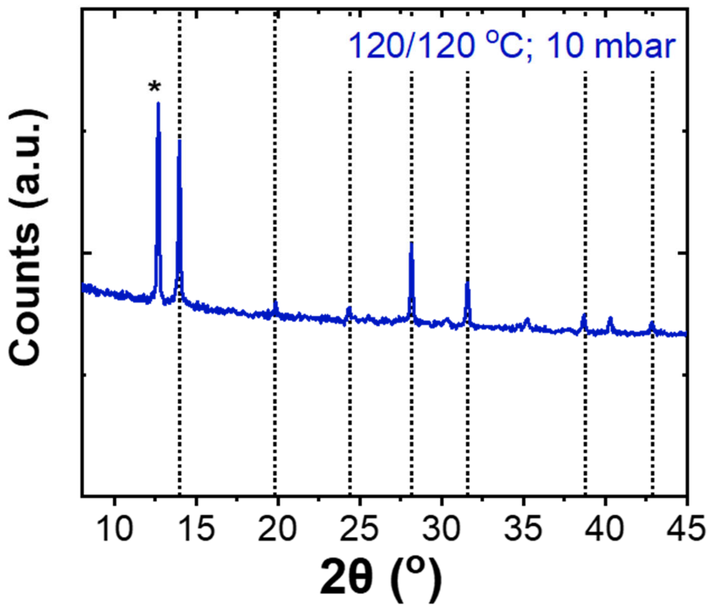

Figure S6. Conversion of inorganic precursor layers (460 nm) at a substrate/source temperature of 120/120 °C. Films were converted for 140 minutes where clear perovskite diffractions are observed (marked by dotted black lines), but an unconverted PbI_2 diffraction is still present, marked by an asterisk (*).

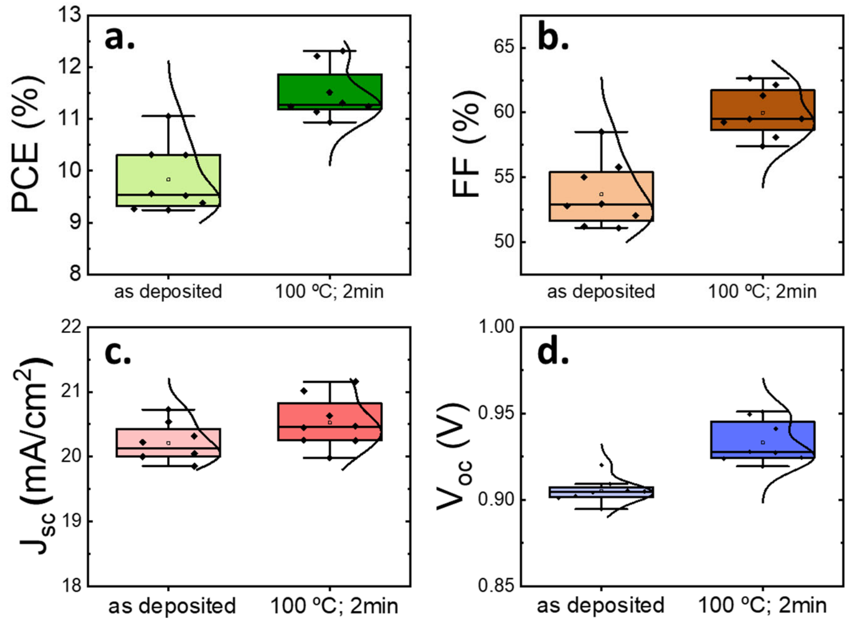

Figure S7. Samples were annealed at 100 ºC for 2 minutes where small improvements in all device parameters were observed. In **a.** the PCE, **b.** fill factor, **c.** short-circuit current density and **d.** V_{oc}.

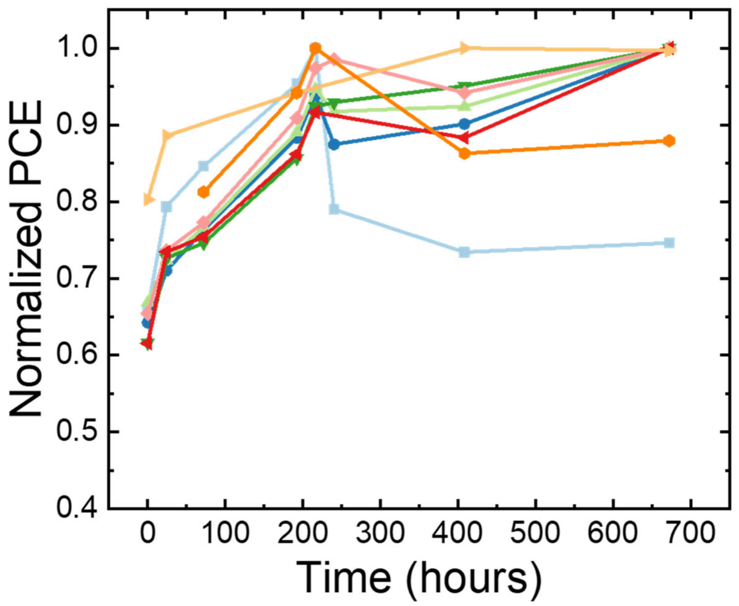

Figure S8. To understand the individual trends of the photoconversion efficiency (PCE) of each device, we plot the normalized PCE over time. Samples were annealed for >650 hours at 85ºC to monitor their stability to thermal stressing. All devices excepting that shown in dark blue exhibit the same trendline displayed in the main text and appear to stabilize after ~200 hours.

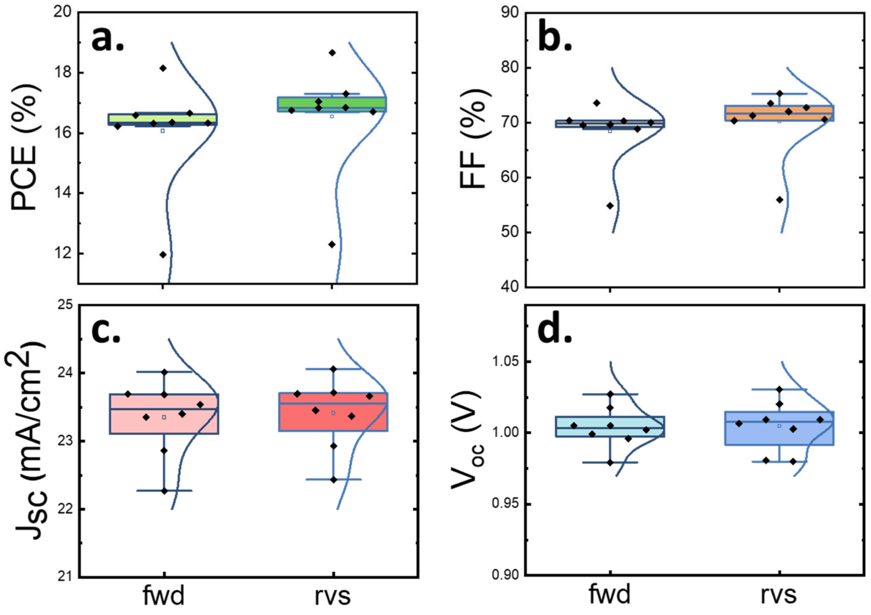

Figure S9. Hysteresis is evaluated by looking at the forward (fwd) and reverse (rvs) JV characteristics of the devices presented in this work (8 samples). Shown in **a.** the photoconversion efficiency (PCE), **b.** the fill factor (FF), **c.** J_{sc} and **d.** V_{oc}. While the reverse characteristics are slightly higher in all 4 parameters, the difference is small with an average PCE increase from 16.07% to 16.55%.

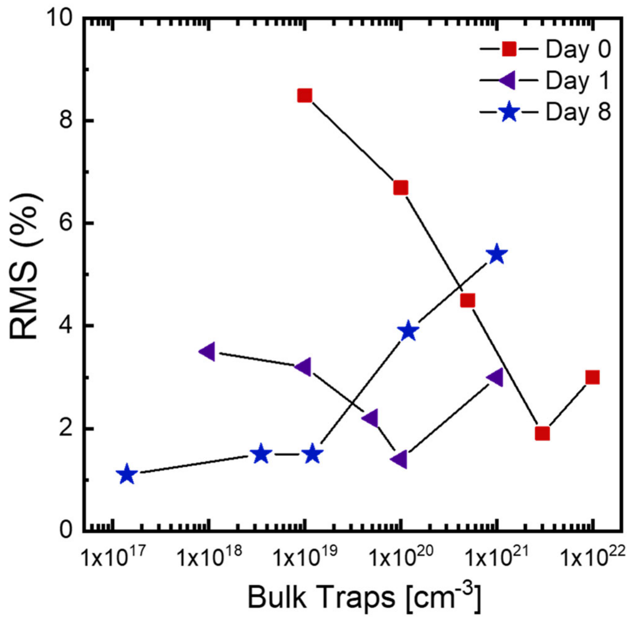

Figure S10. RMS values for the simulated JV curves of a champion device for day 0 (as-deposited), day 1, and day 8 of thermal stressing at 85 ºC in a nitrogen environment. To evaluate the uniqueness of the simulated fit, the RMS is calculated while incrementing the bulk trap density while allowing the surface recombination velocities and carrier mobilities of electrons and holes to vary over a wide range. The minimum RMS for day 0 and day 1 suggest a unique fit for these bulk trap densities. However, for day 8 no minimum is observed, instead plateauing for values <1.1E19 m^{-3}. We suggest this bulk traps no longer limit the performance, instead dominated by interface recombination (associated with surface recombination velocities) and explained in further detail in the main text.

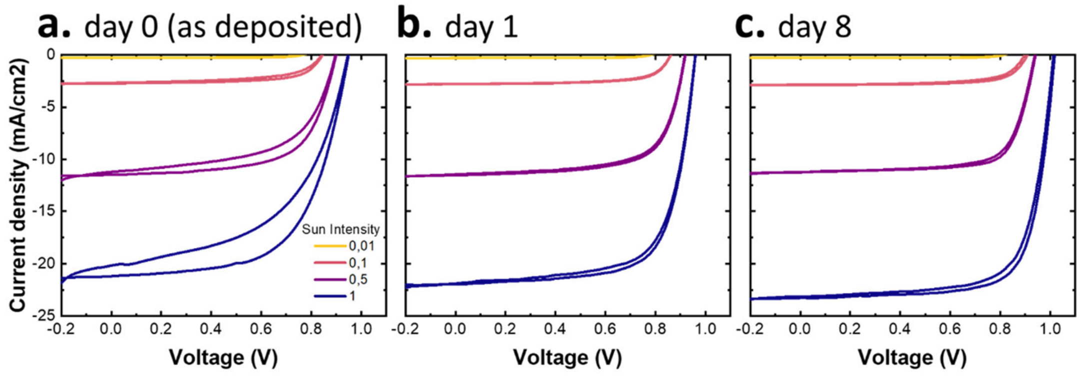

Figure S11. JV parameters at different light intensities (0.01, 0.1, 0.5, and 1 sun) were extracted to evaluate the limiting conditions of the devices. These were tracked for different annealing times **a.** as-deposited **b.** 1 day and **c.** 8 days. The presence of hysteresis in as-deposited devices is clear, which progressively decreases towards longer annealing times.

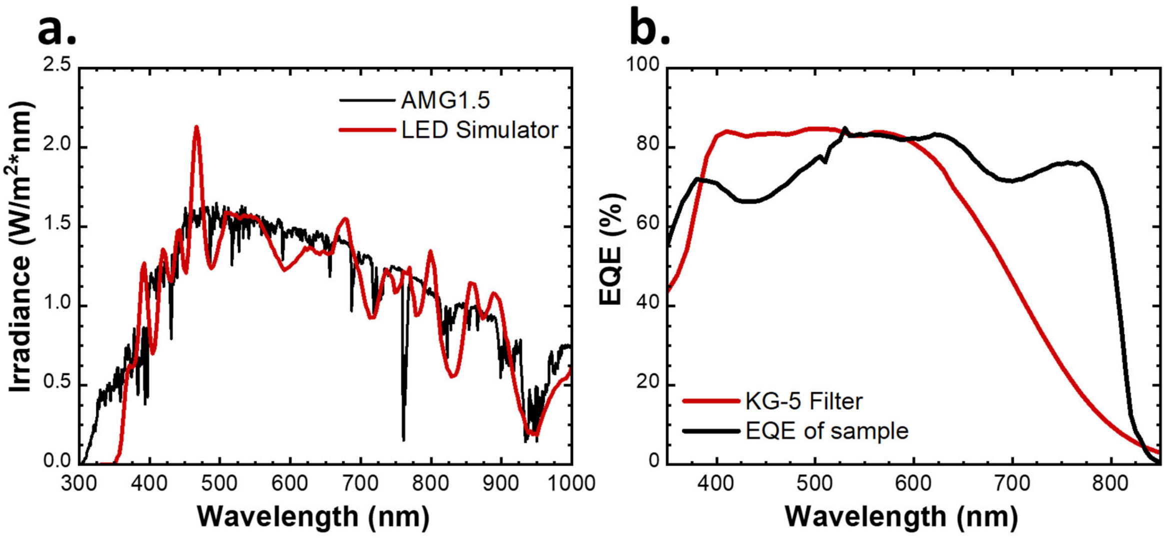

Figure S12. A mismatch factor of 1.01 was calculated using **a.** the spectrum from a Wavelabs Sinus 70 AAA LED solar simulator compared to AMG1.5 and **b.** the external quantum efficiency (%) of one of the solar cells described in this work and a silicon reference diode equipped with a KG-5 filter. This was done using **Eq. S2**.
